# An Improved K-Means Algorithm Based on Evidence Distance

**DOI:** 10.3390/e23111550

**Published:** 2021-11-21

**Authors:** Ailin Zhu, Zexi Hua, Yu Shi, Yongchuan Tang, Lingwei Miao

**Affiliations:** 1 School of Information Science and Technology, Southwest Jiaotong University, Chengdu 611756, China; zhual@my.swjtu.edu.cn; 2 School of Electrical Engineering, Southwest Jiaotong University, Chengdu 611756, China; shiy@my.swjtu.edu.cn (Y.S.); miaolw@my.swjtu.edu.cn (L.M.); 3 School of Big Data and Software Engineering, Chongqing University, Chongqing 401331, China; tangyongchuan@cqu.edu.cn; 4 Qianghua Times (Chengdu) Technology Co., Ltd., Chengdu 610095, China

**Keywords:** k-means clustering, evidence distance, cluster analysis, evidence theory

## Abstract

The main influencing factors of the clustering effect of the k-means algorithm are the selection of the initial clustering center and the distance measurement between the sample points. The traditional k-mean algorithm uses Euclidean distance to measure the distance between sample points, thus it suffers from low differentiation of attributes between sample points and is prone to local optimal solutions. For this feature, this paper proposes an improved k-means algorithm based on evidence distance. Firstly, the attribute values of sample points are modelled as the basic probability assignment (BPA) of sample points. Then, the traditional Euclidean distance is replaced by the evidence distance for measuring the distance between sample points, and finally k-means clustering is carried out using UCI data. Experimental comparisons are made with the traditional k-means algorithm, the k-means algorithm based on the aggregation distance parameter, and the Gaussian mixture model. The experimental results show that the improved k-means algorithm based on evidence distance proposed in this paper has a better clustering effect and the convergence of the algorithm is also better.

## 1. Introduction

With the rapid development of technologies such as cloud computing and the internet of things [1,2], the number of connected devices is increasing and the data generated during human–computer interaction and system operation is growing exponentially [3,4,5]. In response to fast-growing data, data mining technology is constantly updated and iterated [6,7,8]. Clustering is a method of data mining [9]. A data set is divided into multiple clusters through a certain process [10,11]. Data similarity within clusters is high, while data similarity between clusters is low [12,13,14]. Depending on the clustering method and characteristics, clustering algorithms can be classified as: divisional, hierarchical, density algorithms, graph theoretic clustering, grid algorithms, model algorithms, etc. [15,16].

The k-means algorithm has been widely used due to its simple algorithm idea, easy implementation, and high efficiency when processing large-scale data [17,18]. However, the traditional k-means algorithm has major limitations [19,20]. For example, when using Euclidean distance calculations, the degree of discrimination between clusters is low and the output results in unstable values [21,22]. In view of the shortcomings of the traditional k-means algorithm, the k-means algorithm can be improved from different perspectives, such as random sampling, distance optimization, and density estimation methods [23]. Better results can be obtained by improving the method of measuring distances between sample points. Researchers at home and abroad have done a lot of research on distance optimization. Tang et al. [24] proposed the d-k-means algorithm, which weighs the influence of density and distance on clustering based on traditional algorithms, and weights the data. On the basis of weights, the principle of minimum and maximum is introduced to automatically determine the initial cluster centers and the number of centers. Wang et al. [25] proposed an improved k-means algorithm based on distance and sample weights, using dimensionally weighted Euclidean distance to calculate the distance between samples. Wang et al. [26] proposed a new algorithm to help k-means jump out of a local optimum on the basis of several ideas from evolutionary computation, through the use of random and evolutionary processes. Zhao et al. [27] proposed a new variant of k-means. The clustering process is driven by an explicit objective function, which makes the k-means process simpler and converges to a better local optimal solution. Qi et al. [28] proposed an optimized k-means clustering method, named k*-means, and three optimization principles, which can reduce the risk of randomly selecting seeds and reduce the adjustable space. Chen et al. [29] proposed an efficient hybrid clustering algorithm called QALO-K, which combines k-means with an optimized quantum-inspired antlion to make the k-means algorithm converge towards the global optimum. Zhang et al. [30] proposed the DC-k-means algorithm, which added the idea of canopy. At the same time, it combines the sample density in the process of finding the initial clusters, which has a good effect when dealing with low-density areas; however, it is possible that the outliers are classified into one class in the clustering process, which affects the clustering effect.

Dempster–Shafer (DS) theory, also known as evidence theory, was first proposed by Dempster in 1967 and was refined and developed by his student Shafer in 1976. Because evidence theory can meet uncertainty and uncertain information flexibly and effectively without relying on a priori knowledge, it is widely used in many fields, such as: correlation analysis, clustering, classification, etc. Fred et al. discussed the problem of clustering data based on evidence. The n d-dimensional data are decomposed into a large number of compact clusters, and then the k-means algorithm is used to cluster them separately, and several clustering results are obtained, which constitute the association matrix. Finally, the final clustering results are obtained using the MST algorithm on the basis of the association matrix. This method can effectively identify arbitrary clusters in multidimensional data [31]. Li et al. proposed a clustering integration algorithm based on evidence theory, which focuses on the fusion process in the clustering integration algorithm. After obtaining the probability of belonging to each label using the label distribution status of the neighborhood information of the object under test, the probability values are used to form the basic partition. After that, fusion is performed using the Dempster–Shafer fusion rules to obtain the final clustering results. This algorithm avoids blind trust in the obtained labels [32]. Yu et al. proposed a three-way density-peak clustering algorithm based on evidence theory, which uses a density-peak clustering algorithm to obtain clustering centers and noise points, and then uses a mid-distance comparison scheme to merge neighboring points. Finally, the remaining points are assigned using the evidence distance fusion rule. The method effectively solves the problem of error propagation of clustering labels [33].

The main feature of k-means algorithm clustering is the high degree of similarity of data in the same class and the low degree of similarity of data in different classes. The evidence distance in evidence theory can be used to describe the degree of similarity between two bodies of evidence. In order to explore whether a new distance measure can be obtained by using evidence distance instead of Euclidean distance, an improved k-means algorithm based on evidence distance is proposed in this paper. In this paper, we use the attribute values of each sample point to form the evidence body of each sample point, and then select the class in which the cluster center with the smallest distance is added based on the evidence distance from each evidence body to the initial cluster center. Finally, it is divided into k classes to obtain the final clustering results. Through validation on the UCI data set and toy data set, and experimental comparison with the traditional k-means algorithm, and the k-means algorithm based on the aggregation distance parameter and the Gaussian mixture model, the improved k-means algorithm in this paper has better clustering effect and convergence.

The rest of the thesis is organized as follows. The second section provides a review of relevant theory. The third section introduces the algorithmic ideas and motivation of this paper and proposes a k-means algorithm based on evidence distance improvement. The fourth section describes the experimental setting and the chosen algorithm evaluation metrics. The fifth section is devoted to conducting relevant experiments on the UCI dataset and the toy dataset and comparing the experimental results with some existing algorithms. Finally, the sixth section provides the conclusion.

## 2. Related Theories

### 2.1. Traditional K-Means Algorithm

The core idea of the k-means algorithm is: After inputting the k value, randomly select k sample points in the sample point set as the initial clustering center. Then, the distances of the remaining sample points to the initial cluster centers are calculated and the sample points are grouped into the closest clusters. In the generated new clusters, new cluster centroids are reselected and the sample points are clustered and classified again until the clustering classification results no longer change [34]. In the actual application process, after multiple iterations, due to various factors, the termination conditions may not be met. Therefore, a maximum number of iterations will be set in the actual application process, and the calculation will be terminated when the maximum number of iterations is reached. The pseudo-code of the traditional k-means algorithm is summarized as Algorithm 1.
**Algorithm 1** The traditional k-means algorithm.**Input:** data set, k value **Output:** divided into k clustersselect k points from the sample Euclidean from sample point x_i_ to each cluster center**repeat****  for** j=1, 2, ……, m    calculate the Euclidean distance from sample point x_i_ to each cluster center    determine the cluster class mark of x_i_ according to the closest distance    divide the sample points into corresponding clusters  **end for**  calculate new cluster centers**until** the cluster allocation result remains unchanged

The traditional k-means algorithm distance measures include: Euclidean metric, city block distance, Pearson correlation, absolute value correlation, absolute non-central correlation, Spearman rank correlation, and Kendall’s tau. The traditional k-means algorithm mainly uses the Euclidean distance [35]. 

The Euclidean metric [36,37] (also known as the Euclidean distance) is a commonly adopted definition of distance and refers to the true distance between two points in m-dimensional space, or the natural length of a vector (i.e., the distance from that point to the origin). The Euclidean distance in two and three dimensions is the actual distance between two points [38].

The distance measurement formula in two-dimensional space:

(1) $$d=\sqrt{{\left(x_{2}-x_{1}\right)}^{2}+{\left(y_{2}-y_{1}\right)}^{2}}$$

where *d* is the Euclidean distance between the point (*x*_2_, *y*_2_) and (*x*_1_, *y*_1_).

The distance measurement formula in three-dimensional space:

(2) $$d=\sqrt{{\left(x_{2}-x_{1}\right)}^{2}+{\left(y_{2}-y_{1}\right)}^{2}+{\left(z_{2}-z_{1}\right)}^{2}}$$

### 2.2. D-S Evidence Theory

Evidence theory was first proposed by Dempster [39] and further developed by his student Shafer [40], an imprecise reasoning theory, also known as Dempster–Shafer evidence theory. As an uncertain reasoning method, the main characteristics of evidence theory are: it satisfies lower conditions than naive Bayesian probability theory and it has the ability to express ‘uncertainty’ and ‘not knowing’ directly. At the heart of D–S evidence theory is the Dempster combination rule, which integrates the underlying reliability distributions of multiple information sources and obtains a new reliability distribution as an output [41,42,43,44].

**Definition** **1.** *Assuming that a non-empty set Θ is composed of m mutually exclusive events, Θ is the identification frame, Θ = {θ_1_, θ_2_,……θ_n_}. The power set of Θ is represented by 2^Θ^, 2^Θ^ = {∅, {θ_1_}, {θ_1_, θ_2_},……{θ_1_, θ_2_,……θ_n_}} [39,40].*

**Definition** **2.** *For any A* *∈ 2^Θ^, m is the mass function. For any subset A in m, let m(A_i_)* *∈ (0, 1), satisfy the following conditions [39,40]:*

(3) $$\sum_{A\subseteq \Theta }m\left(A_{i}\right)=1,m\left(\Phi \right)=0$$

*Among them, m(A_i_) represents the basic probability of A.*

**Definition** **3.** *Body of Evidence (BOE) is a collection of all focal members and its corresponding mass functions, expressed as follows [39,40]:*

(4) $$\left(B,m\right)=\left\{\left[A,m\left(A\right)\right]|A\epsilon 2^{\theta }\;and\;m\left(A\right)>0\right\}$$

*where B is a subset of the power set2^θ^*.

**Definition** **4.** *A’s belief function (Bel) represents A’s total trust, and A’s likelihood function (Pl) represents the confidence level of not denying A. Belief function (Bel) and likelihood function (Pl) represent the upper limit function and lower limit function of A, respectively, defined as follows [39,40]:*

(5) $$Bel\left(A\right)=\sum_{B\subseteq A}m\left(B\right)\;\;\;\;\;\;\;\forall A\subseteq \Theta$$

(6) $$Pl\left(A\right)=1-Bel\left(\bar{A}\right)=\sum_{B\cap A\neq \emptyset }m\left(B\right)\;\forall A\subseteq \Theta \;$$

*where*  Bel(A)≤Pl(A).

**Definition** **5.** *Assuming that under the basic identification framework, the basic probability distribution functions of the two bodies of evidence (BOE) are m_1_ and m_2_, respectively, the formula for combining according to the Dempster rule is as follows [39]:*

(7) $$m\left(C\right)=m_{i}\left(A\right)⊕m_{i}\left(B\right)=\{\begin{array}{c} 0\;A\cap B=\emptyset \\ \frac{\sum_{A\cap B=C,B\subseteq \Theta }m_{i}\left(A\right)\times m_{i}\left(B\right)}{1-K}\;A\cap B\neq \emptyset \; \end{array}$$

*where* $m_{i}\left(A\right)$, $m_{i}\left(B\right)$ represents two bodies of evidence and m(C) *represents the consensus of two bodies of evidence; K represents the conflicting factor between the two evidence bodies and is defined as follows:*

(8) $$K=\sum_{A\cap B=\emptyset ,\forall A,B\subseteq \Theta }m_{i}\left(A\right)\times m_{i}\left(B\right)$$

**Definition** **6.** *Evidence distance [45,46,47] is usually used to describe the degree of difference between two evidence bodies, and its calculation formula is as follows:*

(9) $$d_{BOE}\left(m_{1},m_{2}\right)={\left(\vec{m_{1}}-\vec{m_{2}}\right)}^{T}\underline{\underline{D}}\left(\vec{m_{1}}-\vec{m_{2}}\right)$$

*The actual calculation formula used is as follows:*

(10) $$d_{BOE}\left(m_{1},m_{2}\right)=\sqrt{\frac{1}{2}{\left(\vec{m_{1}}-\vec{m_{2}}\right)}^{T}\underline{\underline{D}}\left(\vec{m_{1}}-\vec{m_{2}}\right)}$$

*Among them* $d_{BOE}$ *: the distance between the two evidence bodies; m_1_ represents body of evidence 1 and m_2_ represents body of evidence 2;* $\vec{m_{1}}$ *,* $\vec{m_{2}}$ *: the vector constituted by the basic distribution probabilities of the two evidence bodies.* $\underline{\underline{D}}$ *is a 2^N^ × 2^N^ matrix, the row index corresponds to* $\;m_{1}$ *, and the column index corresponds to* $m_{2}$ *, indicating the similarity between the two evidence bodies. Each element of the matrix can be represented as:*

(11) $$d_{ij}=\left|m_{1}\cap m_{2}\right|÷\left|m_{1}\cup m_{2}\right|$$

## 3. Algorithm Design

### 3.1. Algorithm Idea Description

The algorithmic idea is that since the selection of *k* values is not optimized in this method, trial and error is used to find the optimum number of clustering centers, i.e., *k* values. *k* sample points are randomly selected as the initial clustering centers. The attributes of the sample points can be regarded as experts for judging the sample points that belong to a certain class, so the values of the attributes of the sample points are used to form the evidence body of each sample point. After that, the distance from the evidence body to the initial clustering center is calculated using the evidence distance formula. After the initial division of sample points, the clustering centers are then re-selected using the arithmetic mean algorithm. Finally, iterative calculations are performed until the clustering centers do not change.

### 3.2. Algorithm Flow

Step 1: For a given data set, randomly select *k* data sample points as the initial cluster center.

Step 2: Use the attribute value of each sample point to form the evidence body of each sample point.

Step 3: Use Formula (9) to calculate the evidence distance from each sample point to each initial cluster center, select the center with the smallest distance, and add the cluster center to the class.

Step 4: Select *k* cluster centers again.

Step 5: Determine whether the clustering center has changed, if it has changed, continue the iteration, if it remains the same, output the corresponding clustering result.

**Figure 1:**
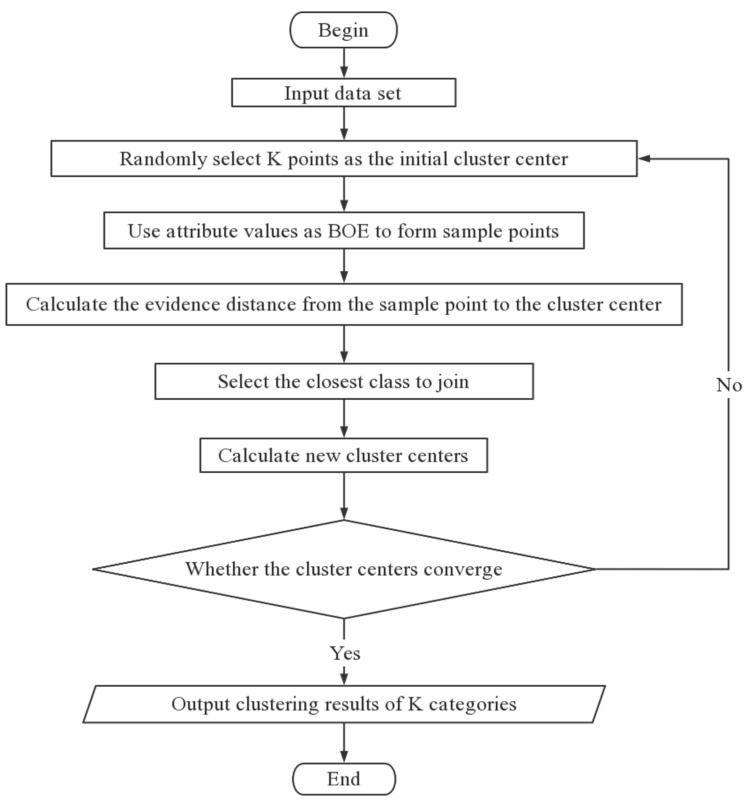
The algorithm flowchart of the improved k-means algorithm.

The algorithm flow chart is shown in Figure 1.

The pseudo code for the evidence distance-based k-means algorithm proposed in this paper is summarized in Algorithm 2.
**Algorithm 2** the k-means algorithm based on evidence distance.**Input:** data set, k value **Output:** clustering results1initialize k cluster centers2use the attribute value of the sample point to construct the BOE of the sample point3**While** true4   num = 0;5   **for** *i* = 0 to k6     *C_i_* = ∅7   **end for**8   **for** *j* = 1 to *m*9     **for** *i* = 1 to k10      calculate evidence distance, *d* = $\sqrt{\frac{1}{2}{\left(\vec{m_{i}}-\vec{m_{j}}\right)}^{T}\underline{\underline{D}}\left(\vec{m_{i}}-\vec{m_{j}}\right)}$11    **end for**12    min = *d*13    **for** *i* = 2 to k14      **if** *d_ij_* < min15         min = *d_ij_*16         temp = *i*17       **end if**18     **end for**19     Lambda = temp20     C(Lambda) = C(Lambda) + {*x_j_*}21   **end for**22   **for** *i* = 1 to k23     *U_i_’* = Update the mean vector based on the previous cluster24     **if** *U_i_’*! = *U_i_*25        *U_i_* = *U_i_’*26     **else**27       num++28     **end if**29   **end for**30   **if** num = *k*31     break32   **end if**33**end while**

## 4. Experiment

### 4.1. Experiment Preparation

The experimental environment is: AMD A10-7300 processor, AMD Raden R7 M260DX graphics card, 8G of running memory, windows10 operating system, and programming with Python 3.7–32 bits.

#### 4.1.1. Experimental Data Set

The data set used in this article comes from the UCI data set. The name of the data set and its attributes are shown in Table 1.

#### 4.1.2. Experimental Evaluation Indicators

The evaluation indicators used in this paper mainly include adjusting the Rand index, the contour coefficient, and the number of iterations. Adjustment of the Rand index and silhouette coefficient are used to evaluate the clustering performance of the algorithm, and the number of iterations is used to evaluate the convergence of the algorithm.

(1) Adjusted Rand index

In the clustering model, assuming that the actual category information is *C* and the clustering result is *K*, a denotes the number of pairs of elements that are both in the same category in *C* and *K*, and b denotes the number of pairs of elements that are both in different categories in *C* and *K*. The Rand index is defined as:

(12) $$RI=\frac{a+b}{C_{2}^{n_{samples}}}$$

where $C_{2}^{n_{samples}}$ represents the total number of pairs of elements that can be composed in the data set. The range of *RI* is [0, 1] and a higher value of *RI* means that the clustering results match the real situation.

The problem with the Rand index is that for two random divisions, the value of the Rand coefficient is not a constant close to zero. Therefore, the adjusted Rand index is used, which has a higher degree of discrimination. The *ARI* is calculated as:

(13) $$ARI=\frac{\left(RI-E\left[RI\right]\right)}{max\left[RI\right]-E\left[RI\right]}\;$$

where *RI* is the Rand index and E[RI] represents the mean value. The range of values for *ARI* is [–1, 1]. A larger value for *ARI* means that the clustering results match the real situation.

(2) Silhouette Coefficient

The silhouette coefficient is a way of evaluating how well clustering works. It was first proposed by Peter J. Rousseeuw in 1986. It combines both cohesion and separation factors. 

Suppose we have completed clustering by some clustering algorithm. For any one of these samples, *A* represents the average distance between the sample and the other samples in its cluster, and *B* represents the average distance between the sample and the samples in the other clusters, the silhouette coefficient of the sample is:

(14) $$S=\frac{B-A}{max\left(A,B\right)}$$

where *S* denotes the silhouette coefficient of a single sample. The total silhouette coefficient of clustering is the average value of all sample silhouette coefficients. The contour coefficients range from (−1, 1), with values closer to 1 indicating better clustering performance, and conversely, values closer to −1 indicating worse clustering performance.

(3) Number of iterations

Number of iterations: how many times the algorithm iterates until the algorithm converges in the actual operation. Since it is a random result, the experiments in this paper take the arithmetic average of the number of iterations after several iterations. The smaller the value, the faster the convergence of the algorithm.

(15) $$\mathrm{Calculation}\;\mathrm{formula}:\;Number\;of\;iterations=\frac{Total\;number\;of\;iterations}{Total\;number\;of\;runs}$$

### 4.2. Experimental Procedure

(1) Import the iris data set and enter the cluster category *k* value.
(2) The traditional k-means method and the improved k-means method are used for clustering, respectively.
(3) Perform clustering 10 times, find the average value, and output the ARI, contour coefficient, and number of iterations as the final result.
(4) Compare the experimental results of the improved algorithm and the traditional algorithm.
(5) Use wine, breast cancer, and other data sets for verification.

## 5. Results and Analysis

### 5.1. Iris Data Set Test Results

**Figure 2:**
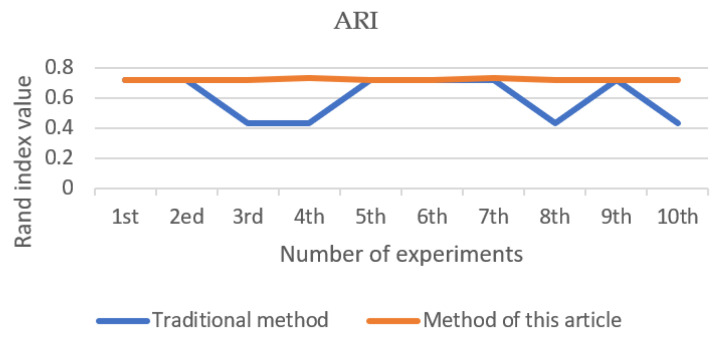
Adjusted rand index (iris).

**Figure 3:**
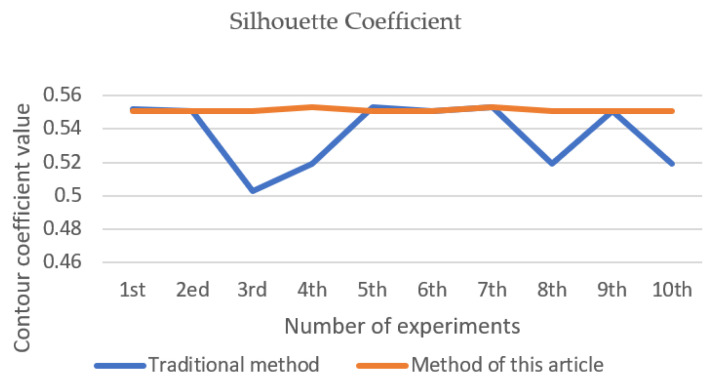
Silhouette Coefficient (iris).

**Figure 4:**
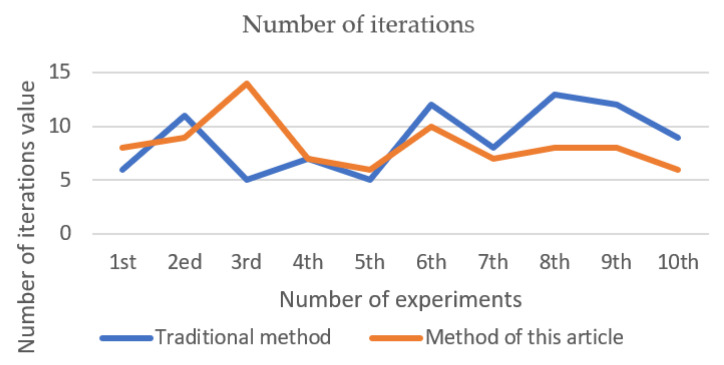
Number of iterations (iris).

After 10 clusters, the *ARI* value of each cluster is shown in Figure 2, the Silhouette Coefficient value is shown in Figure 3, and the number of iterations is shown in Figure 4. The final result is obtained by calculating the average value. The *ARI* value of the traditional method is 0.603, the profile coefficient value is 0.5371, and the number of iterations is 8.8 times. The *ARI* value of the improved method is 0.719, the silhouette coefficient value is 0.5514, and the number of iterations is 8.3 times. From Figure 2, Figure 3 and Figure 4, it can be seen that the new method adopted in this paper is more stable than the traditional method, and the *ARI* value and the silhouette coefficient have been effectively improved. Therefore, the accuracy of this method is better than that of the traditional method, and better clustering effect can be obtained. The improved method has generally reduced the number of iterations compared with the traditional method, so the convergence of the new method is also better than that of the traditional method. 

### 5.2. Validation Results of Other Data Sets

**Figure 5:**
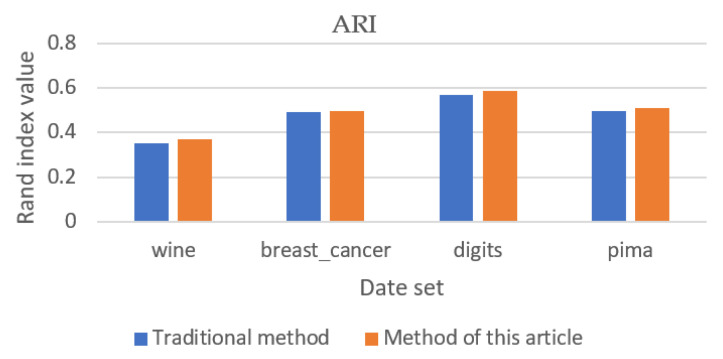
Adjusted rand index (validation data set).

**Figure 6:**
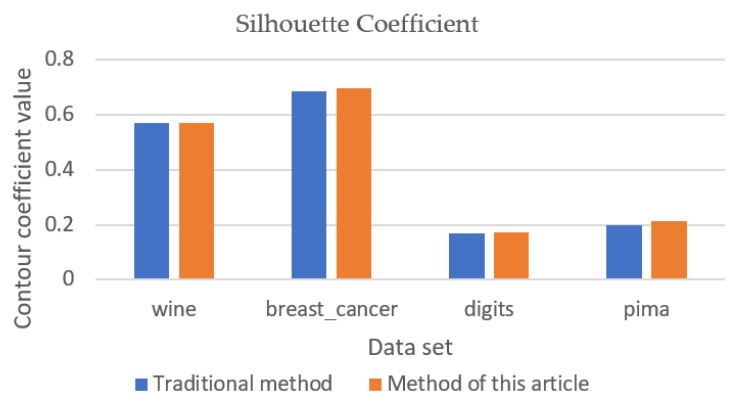
Silhouette coefficient (validation data set).

**Figure 7:**
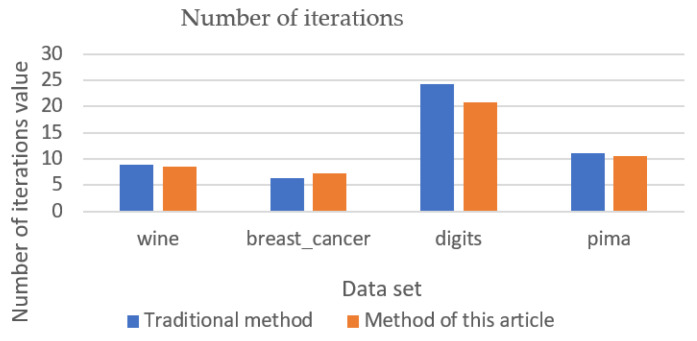
Number of iterations (validation data set).

The clustering effect and convergence of the algorithm were verified by using wine, breast cancer, digits, and pima datasets with ARI values, silhouette coefficient, and number of iterations as shown in Figure 5, Figure 6 and Figure 7. The analysis of Figure 5 and Figure 6 shows that the new method can obtain better clustering results and the output results of the new method are more stable in the output process. However, when there are more attribute values in the data set, the improvement of the new method is smaller. Through the analysis of Figure 7, it can be seen that, except for clustering using the breast cancer data set, the convergence of the new method is slightly worse than that of the traditional method, and the overall convergence of the new method is better than that of the traditional method.

In summary, the new method used in this experiment can obtain better clustering results than traditional methods, and in the output process, the variance between the results is smaller and the output is more stable. At the same time, the convergence of the algorithm is improved to a certain extent.

### 5.3. Algorithm Comparison

**Figure 8:**
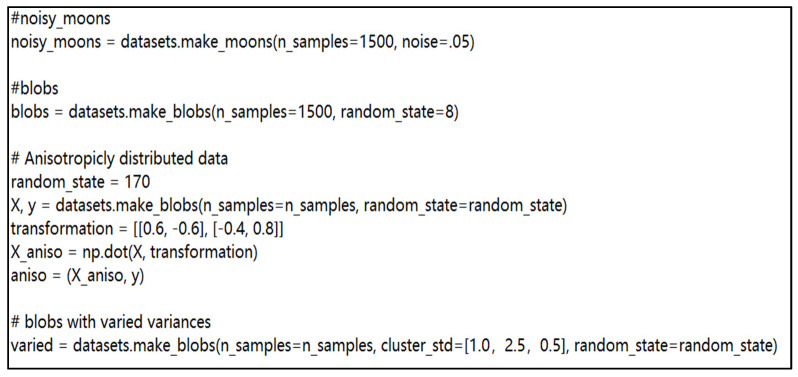
Data set parameters.

In order to conduct a more in-depth verification of the performance of the evidence-distance-based improved k-means algorithm proposed in this paper, the performance of the traditional k-means algorithm (T-K-means), the k-means algorithm based on aggregated distance parameters (AD-K-means) [48], the Gaussian mixture model (GMM) [49], and the k-means algorithm based on evidence distance proposed in this paper (ED-K-means) were selected for experimental comparison. The datasets used for the experiments were the UCI dataset and four toy datasets, iris, digits, wine, noisy-moon, blobs, anisotropicly distributed data, and blobs with varied variances, in that order. The parameters for the four toy datasets are shown in Figure 8. 

**Figure 9:**
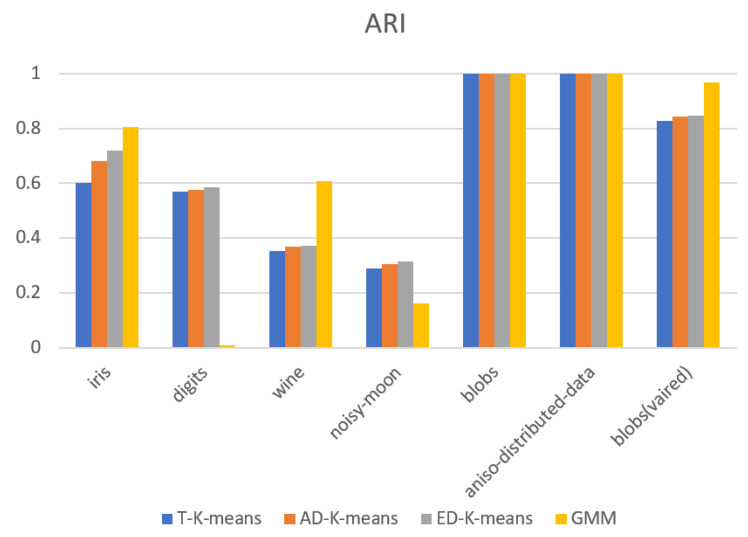
Adjusted rand index.

**Figure 10:**
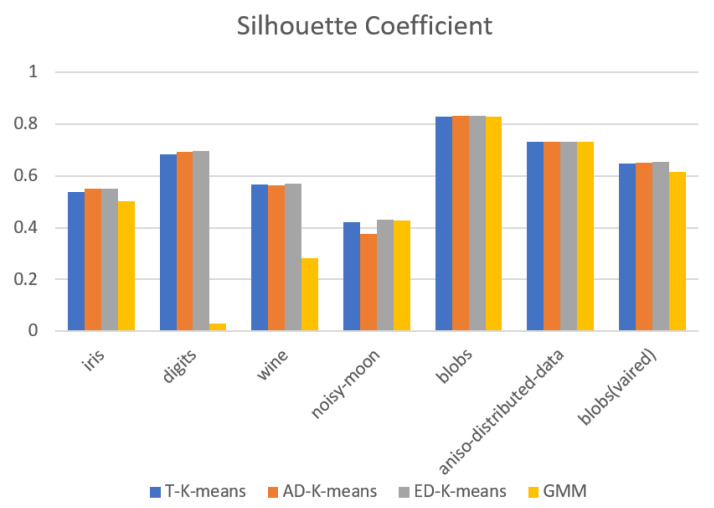
Silhouette coefficient.

**Figure 11:**
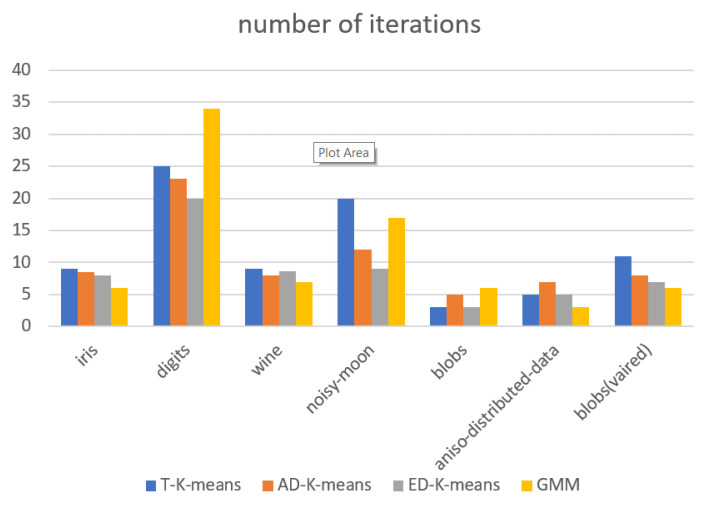
Number of iterations.

**Figure 12:**
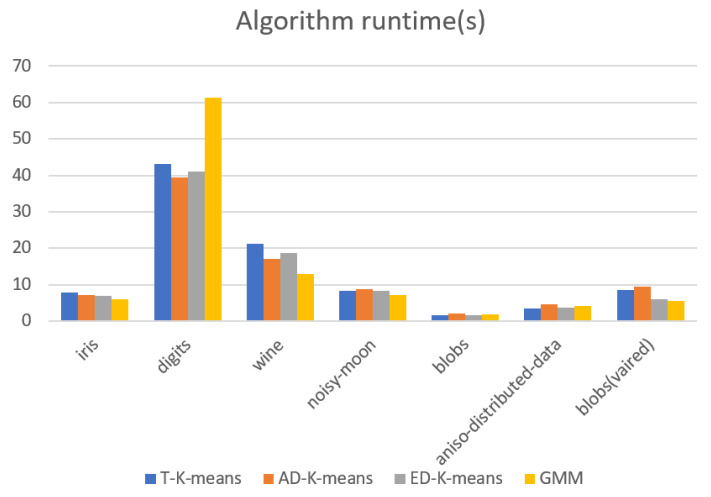
Algorithm runtime.

The experimental results were evaluated in terms of adjusted Rand index (*ARI*), silhouette coefficient, number of iterations, and algorithm runtime. The experimental results are shown in Figure 9, Figure 10, Figure 11 and Figure 12.

Figure 9 shows the results of the adjusted Rand index, with larger values indicating that the clustering results are more consistent with the actual situation. The ED-K-means algorithm proposed in this paper gives higher results than the other three algorithms in both the digits and noisy-moon datasets. In the iris, wine, and blobs with varied variances datasets, the results are slightly lower than those of the GMM algorithm, but higher than those of the other two algorithms. Figure 10 shows the values of the silhouette coefficient, with larger values indicating that the clustering results are more consistent with the actual situation. The results of the ED-K-means algorithm proposed in this paper outperformed the other three algorithms on both the toy dataset and the UCI dataset. Therefore, the ED-K-means algorithm proposed in this paper can achieve better clustering results.

Figure 11 shows the value of the average number of iterations of the algorithm and Figure 12 shows the algorithm running time. Both algorithm metrics are smaller indicating better convergence of the algorithm. The analysis of the results in this figure shows that in the toy dataset, the ED-K-means algorithm proposed in this paper has the lowest number of iterations and the running time is comparable with T-K-means and slightly higher than the GMM algorithm. In the three UCI datasets, the number of iterations is less and the running time is better than that of T-K-means and GMM, and similar to that of AD-K-means. Therefore, on balance, the ED-K-means algorithm proposed in this paper has better convergence.

## 6. Conclusions

In the era of big data, data is expanding, so the clustering algorithm has a wide range of application scenarios. This paper presents an improved k-means algorithm based on evidence distance. The algorithm uses the attribute values of the sample points to form the body of evidence for the sample points. Then, the distance measure between sample points is performed using the evidence distance instead of the Euclidean distance. Finally, the k-means algorithm was used to cluster. Through experimental comparison, the improved k-means algorithm based on evidence distance proposed in this paper has good clustering effect and convergence. However, the initial clustering centers are still selected randomly when processing the data in this paper, so it can be further optimized.

## Figures and Tables

**Table 1:** Experimental data set.

Data Set	Number of Samples	Feature Number	Number of Categories
Iris	150	4	3
Wine	178	13	3
Breast_cancer	699	10	2
Digits	1797	5	9
Pima	768	8	2

## Data Availability

All relevant data are within the paper.
